# Reflections on the study "Central sterile supply department management on hospital-associated infections: a systematic review and meta-analysis" by Shuai *et al*.

**DOI:** 10.1590/S1678-9946202668011

**Published:** 2026-02-02

**Authors:** Eliane Molina Psaltikidis

**Affiliations:** 1 Associação Paulista de Epidemiologia e Controle de Infecções Relacionadas à Assistência à Saúde, São Paulo, São Paulo, Brazil; 2 Universidade Estadual de Campinas, Faculdade de Enfermagem, Grupo de Pesquisa Interdisciplinar em Infecções Relacionadas à Assistência à Saúde, Campinas, São Paulo, Brazil

Dear Editor,

The processing of medical devices (MDs) is globally recognized as a cornerstone of healthcare-associated infection (HAI) prevention, particularly for surgical site infections^1-3^. Advances in surgical technology have increased the demand for complex critical instruments, requiring Central Sterile Supply Departments (CSSDs), to maintain appropriate infrastructure, technology, specialized staff, and robust quality management systems^4^.

The literature documents numerous infection outbreaks in which the causal factor was strongly linked to failures in instrument reprocessing^5-11^. Under routine conditions, however, attributing HAIs to MD processing remains challenging because of their multifactorial nature. In this context, the recent systematic review and meta-analysis published in this journal by Shuai *et al*.^12^ is of great interest to specialists in infection prevention and instrument reprocessing. The authors aimed to evaluate the relationship between CSSD management practices and HAI incidence, also including as a primary outcome various adverse events such as reprocessing failures, delivery delays, instrument loss, and occupational accidents. After screening, eight studies published between 2018 and 2023 were included and four of these (Chen *et al*.^13^, Jing *et al*.^14^, Kong *et al*.^15^ and Xu *et al*.^16^) were incorporated into the HAI meta-analysis.

When examining the forest plots, the unusually high event counts reported for the studies by Chen *et al*.^13^ and Jing *et al*.^14^ raised concerns and prompted verification of the original data. In the study by Chen *et al*.^13^, which implemented a Plan-do-check-action (PDCA)-based quality-improvement strategy combined with surgical center risk management, with the implementation of several improvements, including those related to instrument processing, six surgical site infections occurred in the intervention group versus 17 in controls.

In the meta-analysis by Shuai *et al*.^12^, however, these values appear as 138 versus 146 "infections," although the original publication clearly indicates that these numbers refer to the *rate of adequate surface disinfection* in the operating room. Similarly, Jing *et al*.^14^ conducted a retrospective assessment of conformity with MD reprocessing protocols before and after implementing a sensitive-quality-index system. Although the authors introduced multiple workflow improvements, *infection* was not an outcome measure. The values used by Shuai *et al*.^12^ (239 vs. 268) represent the number of sterilization cycles compliant with institutional protocol, not HAI cases.

The study by Kong *et al*.^15^ conducted in a hospital endoscopy unit, also implemented PCDA-based improvements in the organization of care, staff training, hand hygiene for professionals, and in the sterilization process for endoscopy equipment. Five HAIs were reported among 512 intervention-period patients compared with 14 among 508 controls. However, the article does not specify the types of HAIs, diagnostic criteria, or whether endoscope reprocessing occurred in the endoscopy unit itself (as is common worldwide) or within the hospital CSSD.

In the study by Xu *et al*.^16^ PDCA- and risk-management strategies were applied to nursing care in an hospital endoscopy unit. Four infections occurred among 90 control group patients, compared with one case among 156 intervention group, but again no infection type or diagnostic criteria were provided. All infection-related results were presented alongside air-quality data from the endoscopy room, suggesting that their primary concern may have been airborne transmission.

For the meta-analysis of adverse events, Shuai *et al*.^12^ included data from Chen *et al.* ^13^, Kong *et al*.^15^ and Yang *et al*.^17^. Chen *et al*.^13^ reported a heterogeneous of irregular events which were not limited to reprocessing such as hand-hygiene failures and improper waste disposal (15 events in controls, five in the intervention group). Kong *et al*.^15^ reported 29 occupational accidents in controls versus four in the intervention group.

Yang *et al*.^17^ conducted a controlled study in which CSSD personnel were randomized to routine practice (control) or a defect management improvement mode aligned with Joint Commission International standards (intervention). Adverse events, including distribution errors, instrument damage, sharps injuries, and nosocomial infections totaled 145 in the control group versus 20 in the intervention group. These aggregated totals were used by Shuai *et al*.^12^ in the adverse-event meta-analysis. However, Yang *et al*.^17^ also reported HAI occurrences separately: 29 HAIs in the control group versus two in the intervention group. These data could, and arguably should have been included in Shuai *et al*.^12^'s HAI-focused meta-analysis.

Another source of heterogeneity stems from combining studies conducted in endoscopy units with those examining comprehensive CSSD work processes. CSSDs manage a broad range of complex critical instruments from multiple surgical specialties, requiring more extensive infrastructure, equipment, workflow management, and staff training than endoscopy units. Although endoscope reprocessing is technically challenging and failures have been associated with multiple infection outbreaks^18,19^, the physical layout, workflow, and staffing structure of endoscopy-based reprocessing areas differ substantially from those of hospital-wide CSSDs, limiting comparability.

Accurate data extraction from primary studies is a critical step in systematic reviews and meta-analyses. Misinterpretation or transcription errors can lead to incorrect effect estimates and misleading conclusions. Therefore, methodological guidelines recommend data extraction by two independent reviewers with expertise in both the clinical topic and evidence-synthesis methodology^20^.

Despite the limitations identified in Shuai *et al*.^12^, analyses of this nature are essential for strengthening the international evidence base regarding the relationship between proper MD reprocessing and HAI prevention. Continued refinement of methodological rigor will be crucial as the field advances.

## Data Availability

The complete anonymized dataset supporting the findings of this study is included within the article itself.
